# Additional molecular testing of saliva specimens improves the detection of respiratory viruses

**DOI:** 10.1038/emi.2017.35

**Published:** 2017-06-07

**Authors:** Kelvin KW To, Lu Lu, Cyril CY Yip, Rosana WS Poon, Ami MY Fung, Andrew Cheng, Daniel HK Lui, Deborah TY Ho, Ivan FN Hung, Kwok-Hung Chan, Kwok-Yung Yuen

**Affiliations:** 1 State Key Laboratory for Emerging Infectious Diseases, Li Ka Shing Faculty of Medicine, The University of Hong Kong, Hong Kong, China; 2 Carol Yu Centre for Infection, Li Ka Shing Faculty of Medicine, The University of Hong Kong, Hong Kong, China; 3 Research Centre of Infection and Immunology, Li Ka Shing Faculty of Medicine, The University of Hong Kong, Hong Kong, China; 4 Department of Microbiology, Li Ka Shing Faculty of Medicine, The University of Hong Kong, Hong Kong, China; 5 Department of Microbiology, Queen Mary Hospital, Hong Kong, China; 6 Li Ka Shing Faculty of Medicine, The University of Hong Kong, Hong Kong, China; 7 Department of Medicine, Li Ka Shing Faculty of Medicine, The University of Hong Kong, Hong Kong, China

**Keywords:** influenza, nasopharyngeal, oral fluid, respiratory virus, saliva, viral load

## Abstract

Emerging infectious diseases in humans are often caused by respiratory viruses such as pandemic or avian influenza viruses and novel coronaviruses. Microbiological testing for respiratory viruses is important for patient management, infection control and epidemiological studies. Nasopharyngeal specimens are frequently tested, but their sensitivity is suboptimal. This study evaluated the incremental benefit of testing respiratory viruses in expectorated saliva using molecular assays. A total of 258 hospitalized adult patients with suspected respiratory infections were included. Their expectorated saliva was collected without the use of any special devices. In the first cohort of 159 patients whose nasopharyngeal aspirates (NPAs) tested positive for respiratory viruses during routine testing, the viral load was measured using quantitative reverse transcription PCR. Seventeen percent of the patients (27/159) had higher viral loads in the saliva than in the NPA. The second cohort consisted of 99 patients whose NPAs tested negative for respiratory viruses using a direct immunofluorescence assay. Their NPA and saliva specimens were additionally tested using multiplex PCR. In these patients, the concordance rate by multiplex PCR between NPA and saliva was 83.8%. Multiplex PCR detected viruses in saliva samples from 16 patients, of which nine (56.3%) had at least one virus that was not detected in the NPA. Decisions on antiviral or isolation precautions would be affected by salivary testing in six patients. Although NPAs have high viral loads and remain the specimen of choice for most patients with respiratory virus infections, supplementary molecular testing of saliva can improve the clinical management of these patients.

## INTRODUCTION

Viruses play important roles in respiratory tract infections.^1, 2, 3, 4^ Recently, several novel respiratory viruses have emerged, including the 2009 pandemic influenza virus A(H1N1)pdm09,^5^ the avian influenza viruses A(H7N9) and A(H5N6),^6, 7, 8^ and the Middle East Respiratory Syndrome (MERS) coronavirus.^9^ The novel avian influenza viruses and the MERS coronavirus are associated with high mortality rates of over 30%.

Prompt and accurate detection of respiratory viruses is important for guiding antiviral treatment.^10^ For influenza virus infection, earlier administration of neuraminidase inhibitors is associated with faster resolution of symptoms in randomized clinical trials and improved survival in retrospective studies.^11, 12^ Hyperimmune intravenous immunoglobulin confers survival benefit for severe influenza only if administered within 5 days after symptom onset.^13^ Antibiotic usage can be reduced if a respiratory virus is detected without evidence of bacterial infection.^14^ Early detection is also important for infection control and public health measures.

Nasopharyngeal aspirate (NPA), nasopharyngeal swabs (NPS) (including flocked swabs), and nasal or throat swabs/washes are the recommended upper respiratory tract specimen types for diagnostic testing of respiratory viruses.^15, 16^ Nasopharyngeal specimens are usually considered to have the highest detection rate for respiratory viruses. Nasopharyngeal specimens are often used as the only specimen type in routine clinical practice and in many surveillance studies for the detection of respiratory viruses.^17^ However, in studies that have tested multiple specimen types, nasopharyngeal specimens have been found to be negative in some patients with respiratory virus infections.^18, 19, 20, 21, 22^ Sputum or other lower respiratory tract specimens may contain a higher viral load for some patients, which will improve the detection of viruses. Jeong *et al* showed that nasopharyngeal swabs were negative in 25% of adult patients with sputum positive for respiratory viruses.^19^ However, many patients do not have sputum production or cannot expectorate good quality sputum. Additionally, the collection of tracheal or bronchial specimens involves invasive procedures that are associated with significant discomfort and risk to the patient and pose a risk to healthcare workers.^23, 24^

Saliva can be easily provided by patients without any invasive procedures. However, saliva is rarely used for the detection of respiratory viruses, because it is generally considered to have inferior sensitivity compared with other respiratory tract specimens. MUC5B, salivary gp-340, histatins, and human neutrophil defensins in saliva have been shown to neutralize influenza A virus.^25^ Moreover, saliva is not a suitable specimen type for direct immunofluorescence assay (DFA) because it does not contain a sufficient amount of infected respiratory epithelial cells. Recently, there has been renewed interest in using saliva for the detection of respiratory viruses with molecular assays.^18, 26, 27, 28, 29^ Some of these studies showed that the detection rate of respiratory viruses was actually higher for saliva than for nasopharyngeal specimens.^18^ However, these studies were conducted either in children^28^ or among adult patients with mild symptoms who did not require hospitalization.^18, 27^ Some studies only evaluated the detection of influenza virus.^26, 29^ Furthermore, many of these studies used special collection devices that are not usually available in most hospitals. The utility of saliva for the detection of respiratory viruses among hospitalized adult patients has not been comprehensively studied.

The current study sought to evaluate the benefit of testing expectorated saliva in addition to NPA among adult patients hospitalized for a suspected respiratory tract infection. The aim of the first part of the study was to assess the difference in the viral load between NPA and saliva using quantitative PCR with reverse transcription (RT-PCR). The aim of the second part of the study was to evaluate the incremental benefit of testing saliva in addition to NPA using a commercially available molecular assay. As the current study was designed to enable the easy application of our saliva specimen collection method to any clinical setting, we asked the study participants to provide saliva specimens by simple expectoration into a sterile bottle without using any special collection devices.

## MATERIALS AND METHODS

### Study design and participants

This study was a 1-year prospective study conducted between 1 March 2015, and 29 February 2016, in Queen Mary Hospital, which is a teaching hospital in Hong Kong with 1600 beds. This study was approved by the Institutional Review Board of the University of Hong Kong/Hospital Authority Hong Kong West Cluster. Written informed consent was obtained from all study participants. Patients were eligible for recruitment if they were hospitalized adult patients aged 18 years or above with a suspected respiratory tract infection and had NPA samples obtained for respiratory virus testing. Saliva specimens were collected from the enrolled patients when the result of the routine clinical testing for respiratory viruses in the clinical microbiology laboratory of Queen Mary Hospital was known. Therefore, a patient’s saliva specimen was always collected after the NPA specimen. Patients were excluded from the study if they were discharged from the hospital before enrollment, were unable to provide a saliva specimen, or refused or were unable to provide written informed consent.

**Figure 1:**
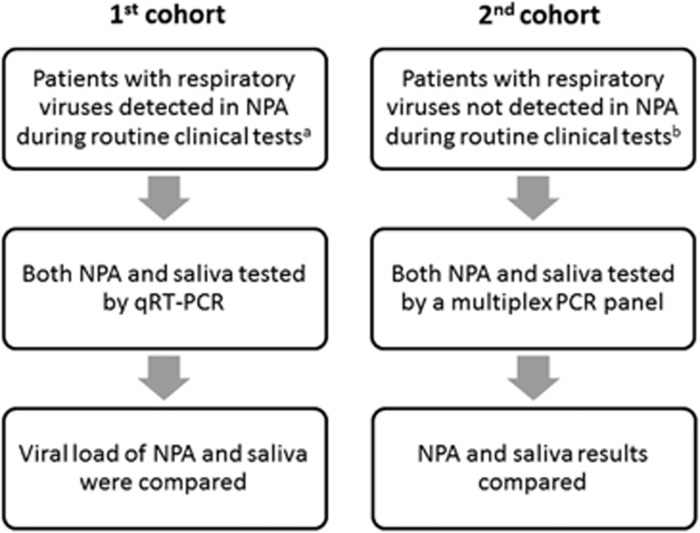
Study design. nasopharyngeal aspirate, NPA; quantitative PCR with reverse transcription, qRT-PCR. ^a^Routine clinical testing was performed using antigen detection by the DFA, which included the influenza A and B viruses, parainfluenza virus types 1–3, respiratory syncytial virus, human metapneumovirus and adenovirus. From 1 March to 8 April 2015 (during the peak influenza A virus season), monoplex real-time RT-PCR for the influenza A M gene was performed for patients admitted to the general medical ward. ^b^Patients whose NPA specimens either tested negative for respiratory viruses by DFA or had insufficient NPCs for DFA during routine clinical testing. Insufficient NPCs is defined as <20 NPCs in the entire well.

The first part of the study consisted of patients whose NPA samples tested positive for respiratory viruses by DFA or the influenza A virus M gene by real-time RT-PCR during routine respiratory virus testing in our clinical microbiology laboratory (Figure 1). The viral load was determined in both the NPA and the saliva samples using quantitative RT-PCR (qRT-PCR). During the first phase (1 March to 15 June 2015), only patients who tested positive for influenza A virus were included. During the second phase (16 June 2015 to 29 February 2016), patients who tested positive for any respiratory viruses were included.

The second part of the study consisted of patients whose NPA specimens either tested negative for respiratory viruses by DFA or had insufficient nasopharyngeal columnar epithelial cells (NPCs) in the NPA sample for DFA during routine clinical testing (Figure 1). Insufficient NPCs was defined as <20 NPCs in the entire well. Multiplex PCR was used to test for respiratory viruses in both the NPA and the saliva samples.

### Data collection

Patient data concerning demographics, underlying diseases, clinical findings and radiological changes were recorded in a predesigned database. The Charlson comorbidity score was calculated.^30^ Pneumonia was defined by radiological evidence of new or increased pulmonary infiltrate in a chest radiograph and at least one of the following symptoms (cough with or without sputum production, dyspnea, tachypnea, or pleuritic chest pain) plus one auscultatory finding or one sign of infection (core body temperature >38 °C, shivers, leukocyte count >10 000 cells/μL or <4000 cells/μL) as described previously.^31^

### Routine respiratory virus testing in the clinical microbiology laboratory

NPA samples were collected in viral transport medium (VTM) as described previously.^32^ Routine testing for respiratory viruses was performed using antigen detection by DFA (D^3^ Ultra 8 DFA Respiratory Virus Screening and Identification Kit, Diagnostic Hybrids, Inc., Quidel, San Diego, CA, USA), which included influenza A virus, and influenza B virus, parainfluenza viruses 1–3, respiratory syncytial virus (RSV), human metapneumovirus (hMPV) and adenovirus. From 1 March to 8 April 2015 (during the peak influenza A virus season in Hong Kong), real-time RT-PCR for the influenza A virus M gene was performed for patients admitted to the general medical ward as described previously.^33^ During this period, DFA was not performed if the NPA samples tested positive for influenza A virus using RT-PCR.

### Saliva specimen collection

Patients were instructed to expectorate saliva into a sterile container and 2 mL of VTM was added to the container in the microbiology laboratory. The volume of saliva ranged between ~0.5 and 1 mL.

### Quantitative RT-PCR for respiratory viruses

Total nucleic acid (TNA) extraction was performed using the easyMAG instrument (bioMerieux, Boxtel, Netherlands) as described previously.^34, 35^ NPA or saliva specimens in VTM (250 μL) were mixed with lysing buffer. After extraction, the nucleic acids were recovered using 55 μL of elution buffer.

qRT-PCR for influenza A virus detection was carried out using Superscript III Platinum One-Step RT-PCR reagents (Invitrogen, Carlsbad, CA, USA) as described previously with modifications.^13, 33, 34^ The reagent mixture (25 μL) contained the 1 × Reaction Mix, Superscript III RT/Platinum Taq Mix, ROX reagent, 0.8 μM of the forward and reverse primers, 0.2 μM of the probe and 5 μL of TNA as the template. The thermal cycling conditions were 30 min at 50 °C for reverse transcription, 2 min at 95 °C for RT inactivation/initial denaturation, and 50 cycles of 15 s at 95 °C and 30 s at 55 °C. All reactions were performed using the StepOnePlus Real-Time PCR System (Applied Biosystems, Foster City, CA, USA).

The qRT-PCR for the detection of influenza B virus, parainfluenza viruses 1–3, hMPV and respiratory syncytial virus was carried out using AgPath-ID One-Step RT-PCR reagents (Applied Biosystems). The reagent mixture (25 μL) contained 1 × RT-PCR Buffer, 1 × RT-PCR Enzyme Mix, 0.4 μM of the forward and reverse primers, 0.12 μM of the probe and 5 μL of TNA as the template. The thermal cycling conditions were 10 min at 45 °C for reverse transcription, 10 min at 95 °C for RT inactivation/initial denaturation, and 50 cycles of 15 s at 95 °C and 45 s at 55 °C. All reactions were performed using the LightCycler 96 Real-Time PCR System (Roche, Basel, Switzerland). The primer and probe sequences are shown in Table 1.

### Multiplex PCR panel for respiratory viruses

The multiplex PCR for respiratory viruses was performed using the NxTAG Respiratory Pathogen Panel (IVD) (Luminex, Austin, TX, USA) according to the manufacturer’s instructions. Respiratory viruses detected by this assay included influenza A virus, influenza B virus, RSV A and B, enterovirus/rhinovirus (EV/RV), parainfluenza viruses 1–4, hMPV, adenovirus, coronaviruses HKU1, NL63, 229E and OC43, and human bocavirus. Extracted TNA was added to pre-plated Lyophilized Bead Reagents. The reaction was amplified via RT-PCR, and the reaction product underwent bead hybridization within the sealed reaction well. The hybridized and tagged beads were sorted and read on the MAGPIX instrument, and the signals were analyzed using the NxTAG Respiratory Pathogen Panel Assay File for the SYNCT Software (Luminex, Austin, TX, USA).

### Statistical analysis

The Mann-Whitney *U-*test and Fisher’s exact test were used for comparisons of continuous and categorical variables, respectively. The Wilcoxon matched-pairs signed rank test was used for the comparison between the NPA and saliva viral loads. Correlations between the viral loads in the NPA and saliva specimens were determined using Spearman’s correlation coefficient. McNemar’s test was used to compare the positivity rates between the NPA and the saliva specimens. Log-transformed data were used for statistical calculations of the viral load. The statistical analysis was performed using SPSS 21.0 (IBM Corp., Armonk, NY, USA) or GraphPad Prism 6.0 (GraphPad Software, La Jolla, CA, USA).

## RESULTS

### First cohort: comparison of the viral loads between the NPA and the saliva samples

**Figure 2:**
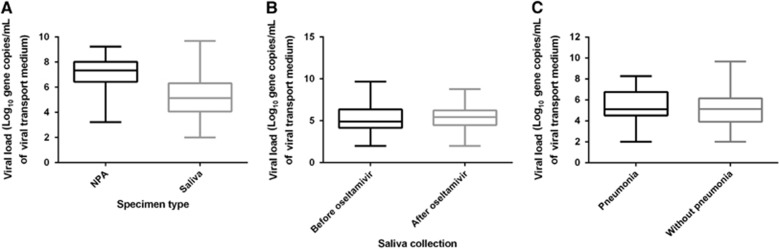
Viral loads in the NPA and the saliva specimens for all patients in the first cohort. The number of patients infected with each of the respiratory viruses is outlined in Table 2. (**A**) Comparison of viral loads between the NPA and saliva specimens. (**B**) Comparison of the saliva viral load of influenza A and influenza B in patients with saliva collected before and after oseltamivir treatment. (**C**) Comparison of the saliva viral loads in patients with or without pneumonia. Medians, quartiles, and ranges are shown. nasopharyngeal aspirate, NPA.

The first cohort consisted of 159 patients with known respiratory virus infection (Table 2 and Supplementary Table S1). The median age was 69 years, with a range from 20 to 98 years. The median Charlson comorbidity score was 1. Forty (25.2%) patients had pneumonia, two patients (1.3%) required admission to the intensive care unit, and two patients (1.3%) died. Most of the saliva specimens were collected <2 days after the collection of the NPA sample (91.8%, 146/159). Among this first cohort of patients with a respiratory virus detected in the NPA sample during routine clinical testing, the qRT-PCR for the same virus was positive in the NPA specimens of all patients and in the saliva specimens of 91.8% (146/159) of the patients (Table 2). Among the 99 patients who tested positive for influenza A virus, the NPA specimens of 12 patients were tested by influenza A virus M gene RT-PCR only during the routine clinical service. The saliva sample was positive by qRT-PCR in 11 out of these 12 patients (91.7%). Although the median viral load was significantly greater in the NPA than in the saliva samples (7.23 vs 5.30 log_10_ copies per mL, *P*<0.0001), 17.0% (27/159) of the patients had a higher viral load in the saliva than in their NPA sample (Figure 2A). No significant correlation was found between the viral load in the NPA and that in the saliva (*P*=0.071). Among the patients with influenza A or influenza B virus infection, no significant difference was found in the median salivary viral load between the patients with saliva collected before and those with saliva collected after oseltamivir treatment (5.43 vs 4.90 log_10_ copies per mL, *P*=0.476; Figure 2B). No significant difference in the median viral load was detected in the saliva specimens between patients with pneumonia and those without pneumonia (5.12 vs 5.13 log_10_ copies per mL, *P*=0.218; Figure 2C).

### Second cohort: multiplex PCR testing of the NPA and the saliva samples

In the second part of the study, multiplex PCR using the Luminex NxTAG Respiratory Pathogen Panel was performed on the saliva and NPA specimens of 99 patients whose NPA specimens tested negative by DFA (*n*=80) or were considered unsuitable for DFA due to insufficient NPCs (*n*=19) during routine clinical testing (Supplementary Table S1). Twenty-five patients (25.3%) had pneumonia. At least one respiratory virus was detected in 22.2% (22/99) of the patients, with one virus detected in 20.2% (20/99) of the patients, two viruses detected in 1.0% (1/99) of the patients, and three viruses detected in 1.0% (1/99) of the patients. Hence, a total of 25 viruses were detected (Tables 3 and 4). At least one respiratory virus was detected in 14.1% (14/99) and 16.2% (16/99) of the NPA and the saliva samples, respectively (Table 3; *P*=0.789). Eighty-three patients (83.8%) showed complete concordance, with the multiplex PCR on the NPA and the saliva samples showing the presence or absence of exactly the same respiratory viruses (κ coefficient of 0.335 (95% confidence interval: 0.081–0.589)).

Respiratory viruses were detected in the saliva specimens of 8 (9.4%) of the 85 patients whose NPA specimens tested negative by NxTAG Respiratory Pathogen Panel, including three patients with influenza A virus, two patients with hMPV, two patients with EV/RV and one patient with coronavirus OC43 (Table 5). For the 14 patients whose NPA specimens tested positive by the NxTAG Respiratory Pathogen Panel, one patient had an additional virus species detected in her saliva (Patient 4 in Table 5). Potential changes in the antiviral treatment or infection control practice would be possible for six of the nine patients with additional viruses detected in their saliva. The three patients with influenza A virus infection could have been given neuraminidase inhibitor, whereas the three patients with hMPV should have been placed under contact precaution.^36^

## DISCUSSION

Novel molecular assays have been developed for the diagnosis of respiratory virus infections,^37, 38, 39, 40, 41^ which have greatly enhanced the sensitivity of the detection of respiratory viruses. However, very few studies have reported an evaluation of specimen types to improve the detection rate. This study evaluated the use of saliva in addition to NPA for the diagnosis of respiratory viral infections in two parts. In the first part of this study, saliva had a higher viral load than NPA in 17.0% of the patients who tested positive for respiratory viruses by DFA or influenza A virus by RT-PCR in their NPA samples. In the second part of this study, saliva specimens tested positive in eight (9.4%) of the 85 patients whose NPA specimens tested negative by multiplex PCR. Among the 14 patients whose NPA specimens were unsuitable for DFA or tested negative by DFA but positive by multiplex PCR, one patient (7.1%) had an additional respiratory virus detected in her saliva specimen using multiplex PCR. Importantly, a change in antiviral treatment or isolation precaution could have occurred in six out of these 9 patients with additional respiratory viruses detected in their saliva.

In some previous studies, saliva was obtained using a dropper^29^ or a special sponge on a stick.^28^ The advantage of using these devices is that the amount of saliva is standardized, and the saliva is less likely to be contaminated by sputum. However, these collection devices are not usually available in a general medical ward setting. Furthermore, the collection of saliva with these devices requires help from healthcare workers. In the present study, we simply asked the patients to expectorate saliva into the standard sterile sputum containers used routinely in our hospital. As no special collection device is required, the use of expectorated saliva can be implemented easily in daily clinical practice. One possible concern is that the expectorated saliva may be mixed with sputum, which may contain a higher viral load than saliva. However, because the aim of using saliva is to increase the detection rate, this perceived limitation is actually favorable.

In real-life clinical practice, testing both NPA and saliva simultaneously is too costly. A more cost-effective approach is to test saliva only if the NPA result is negative. Hence, in the current study, we mimic this situation by collecting saliva only when the NPA result is available. Our previous studies on influenza viruses showed that patients usually had higher viral loads in their NPA upon hospital admission.^33, 34, 42^ Therefore, saliva collected later may have a lower viral load and may result in a lower sensitivity of detection.

Although this study was primarily designed to evaluate the incremental benefit of testing saliva in addition to NPA, the results provided some insights into the potential use of testing saliva alone. Among NPA-positive specimens, the detection rate in saliva was 91.8% (146/159) in the first part of the study and 57.1% (8/14) in the second part of the study. Notably, the detection rate of respiratory viruses by multiplex PCR in the second part of the study was slightly higher in saliva (16.2%) than in NPA (14.1%). Kim *et al* also showed that multiplex PCR of saliva and NPS specimens had similar detection rates.^18^ Therefore, the use of saliva alone may not be inferior to the use of NPA if only a single type of specimen is tested. Testing saliva over NPA has many advantages. First, collecting saliva rather than NPA avoids patient discomfort.^43^ Second, the collection of saliva is suitable for patients for whom the collection of nasopharyngeal specimens is contraindicated, such as patients with severe bleeding tendency. Third, patients can provide saliva specimens after simple instruction, whereas the collection of nasopharyngeal specimens must be performed by healthcare personnel. This approach would reduce the delay in specimen collection. Finally, the collection of saliva does not require any special infection control precautions and can be performed in any clinical setting with standard precautions. By contrast, the procedures for the collection of nasopharyngeal specimens are potentially aerosol-generating and therefore pose significant risks to healthcare workers and other patients. Some health authorities have recommended that nasopharyngeal specimens should be collected in a negative pressure isolation room for patients with suspected MERS coronavirus or novel influenza viruses.^44^

Sputum is also a non-invasive specimen that has been used for the detection of respiratory viruses, especially in patients with pneumonia.^17, 19, 22^ However, many patients with respiratory virus infection do not have sputum production or cannot expectorate good quality sputum. By contrast, saliva specimens can be obtained much more easily than sputum specimens.

DFA is routinely used in our clinical setting for the diagnosis of respiratory virus infections. The advantages of DFA include a relatively low cost, rapid results and simultaneous detection of multiple viral pathogens. However, the sensitivity of DFA is low compared with molecular assays. During the 2009 influenza pandemic, the sensitivity of DFA ranged from 39% to 93%.^5^ DFA also has a low sensitivity for influenza A H7N9 virus infection.^40^ Another disadvantage of DFA is that it is not suitable for saliva specimens, because DFA can only detect viral antigens that are present inside infected cells, which are not present in the saliva. Therefore, to avoid bias between different tests, we used a multiplex PCR panel to test both the NPA and the saliva specimens. Although the multiplex PCR panel used in the second cohort (the NxTAG Respiratory Pathogen Panel) is more sensitive than DFA, it has lower sensitivity than real-time RT-PCR assays. The sensitivity of the NxTAG Respiratory Pathogen Panel ranged from 71.4% to 100% compared with real-time PCR or RT-PCR.^45^ Therefore, some patients with respiratory virus infection may be missed even when NPA and saliva specimens are tested with this multiplex PCR panel.

This study has several limitations. First, our saliva collection method is not suitable for patients who cannot expectorate saliva, such as patients who are unconscious. For these patients, suction aspiration of saliva is required. Second, in the first cohort, we only evaluated viruses included in the routine DFA or influenza A virus RT-PCR. Other important respiratory viruses, such as rhinoviruses and coronaviruses, need to be further evaluated. Third, the number of specimens of each virus species is too small to compare the differences in sensitivity for specific virus species.

Although the viral load in the saliva is lower than the viral load in the NPA for most patients, we have shown that testing both expectorated saliva and NPA can significantly improve the detection of respiratory viruses compared with testing of NPA alone. Saliva should be obtained from patients with a suspected respiratory virus infection but a negative test for respiratory viruses. Furthermore, saliva should be evaluated as the specimen type in a diagnostic testing for novel respiratory viruses. This approach will ultimately lead to improvement in the management of patients and the prevention of community or nosocomial spread of infections.

## Figures and Tables

**Table 1:** Primers and probes used in the quantitative reverse transcription PCR

**Virus**	**Primer/probe sequence (5′-3′)**	
Influenza A virus	Forward	GACCRATCCTGTCACCTCTGAC
	Reverse	AGGGCATTYTGGACAAAKCGTCTA
	Probe	FAM- TGCAGTCCTCGCTCACTGGGCACG -BHQ1
Influenza B virus	Forward	ACAATTGCCTACYTGCTTTCA
	Reverse	TCTTTCCCACCRAACCAAC
	Probe	HEX- AGAAGATGGAGAARGCAAAGCAGAACTAGC -IABkFQ
Human metapneumovirus	Forward	CATAYAARCATGCTATATTRAAAGAGTCTC
	Reverse	CCTATYTCWGCAGCATATTTGTAATCAG
	Probe	FAM- CAACHGCAGTRACACCYTCATCATTRCA -IABkFQ
Respiratory syncytial virus	Forward	CTTAGCAAAGTCAAGTTRAATGATACA
	Reverse	TGCACATCATAATTRGGAGTGTC
	Probe	HEX- ACYATYCAACGKAGYACAGGAGA -IABkFQ
Parainfluenza virus type 1	Forward	GGAGGAGCAATTATACCTGGTCA
	Reverse	TGTATCCARTGAGTGGGCTA
	Probe	LC610- ATTAGGCCCGAGTGTRACRGATGATGC -BBQ
Parainfluenza virus type 2	Forward	TATGCYATGGTGGGAGACATT
	Reverse	GCCATCTTGTTCCAAGTCCAT
	Probe	FAM- CCTCCCATTCCGCTGTGTTCAATRTACTT -IABkFQ
Parainfluenza virus type 3	Forward	AGCTATYACTAGYATCTCAGGGT
	Reverse	CCCAATCTGATCCACTGTGT
	Probe	HEX- TCAGACAAGATGGAACAGTGCAGGCA -IABkFQ

**Table 2:** Detection of viruses using quantitative real-time reverse transcription PCR in the first cohort of patients

**Virus**	**Number of patients with the virus detected in the NPA during routine clinical testing**^a^	**Saliva-positive, number (%)**	**Higher viral load in saliva than in NPA, number (%)**
Influenza A virus	99^b,c^	95 (96.0)	17 (17.2)
Respiratory syncytial virus	18	15 (83.3)	1 (5.6)
Human metapneumovirus	14	11 (78.6)	2 (14.3)
Influenza B virus	12	10 (83.3)	3 (25)
Parainfluenza virus type 3	8	8 (100)	2 (25.0)
Parainfluenza virus type 1	5	4 (80)	1 (20.0)
Parainfluenza virus type 2	3	3 (100)	1 (33.3)
All viruses	159	146 (91.8)	27 (17.0)^d^

Abbreviations: direct immunofluorescence, DFA; nasopharyngeal aspirate, NPA; PCR with reverse transcription, RT-PCR.

a Routine clinical testing was performed using antigen detection by DFA, which included influenza A and B viruses, parainfluenza virus types 1–3, respiratory syncytial virus, human metapneumovirus and adenovirus. From 1 March to 8 April 2015 (during the peak season of influenza A virus), monoplex real-time RT-PCR for influenza A M gene was performed for patients admitted to the general medical ward. Among patients recruited in this study, there were no patients with adenovirus detected in their NPA during routine clinical testing.

b Eighty-four patients were infected with H3 subtype, while 15 patients were infected with H1 subtype.

c Eighty-seven patients (87.9%) were tested positive for influenza A virus using antigen detection by direct immunofluorescence. Twelve patients (12.1% all infected with H3 subtype) admitted to the general medical ward during the peak influenza season were tested by RT-PCR for influenza virus M gene only during the routine clinical testing.

d The denominator includes those patients for whom their saliva specimens were tested negative for respiratory viruses. This is because these patients may have a low quantity of respiratory virus present in their saliva specimens but below the detection limit of the assay.

**Table 3:** Detection of respiratory viruses in nasopharyngeal aspirate and saliva using NxTAG Respiratory Pathogen Panel in the second cohort of patients^a^

	**Number (%) of patients**	
	**All NPA (*n*=99)**	**NPA with sufficient nasopharyngeal epithelial cells (*n*=80)**
Number of patients with ≥1 respiratory virus detected		
NPA or saliva	22 (22.2)	17 (21.3)
NPA	14 (14.1)	12 (15.0)
Saliva	16 (16.2)	12 (15.0)

*Concordant*		
Same respiratory virus detected in both NPA and saliva	6 (6.1)	5 (6.3)
No respiratory viruses detected in NPA or saliva	77 (77.8)	63 (78.8)
Total	83 (83.8)	68 (85.0)

*Discordant*		
Additional respiratory virus detected in saliva	9 (9.1)^b^	6 (7.5)
Additional respiratory virus detected in NPA	7 (7.1)^c^	6 (7.5)
Total	16 (16.2)	12 (15.0)

Abbreviation: nasopharyngeal aspirate, NPA.

a Respiratory viruses detected by NxTAG Respiratory Pathogen Panel include influenza A virus, influenza B virus, respiratory syncytial viruses A and B, enterovirus/rhinovirus, parainfluenza viruses 1-4, human metapneumovirus, adenovirus, coronaviruses HKU1, NL63, 229E and OC43, and human bocavirus.

b For eight patients, respiratory virus was not detected in the NPA by multiplex PCR. For one patient (patient 4 in Table 5), enterovirus/rhinovirus was detected in both NPA and saliva, but human metapneumovirus was detected in saliva only.

c For six patients, respiratory virus was not detected in saliva. For one patient, human metapneumovirus and respiratory syncytial virus A was detected in both NPA and saliva, but coronavirus 229E was detected in NPA only.

**Table 4:** Respiratory viruses detected using multiplex PCR panel in the second cohort of patients^a^

**Respiratory virus**	**Number of patients**			
	**Total^b^**	**NPA and saliva**	**NPA only**	**Saliva only**
Human metapneumovirus	9	3	3	3
Rhinovirus/enterovirus	4	1	1	2
Influenza A	5	1	1	3
Influenza B	2	2	0	0
Respiratory syncytial virus	2	2	0	0
Coronavirus OC43	1	0	0	1
Coronavirus 229E	1	0	1	0
Adenovirus	1	0	1	0
Total	25	9	7	9

Abbreviation: nasopharyngeal aspirate, NPA.

a Multiplex PCR was performed using NxTAG Respiratory Pathogen Panel, which included influenza A and B, influenza A H1, influenza A H3, respiratory syncytial viruses A and B, respiratory syncytial virus B, parainfluenza viruses 1-4, human bocavirus, human metapneumovirus, rhinovirus/enterovirus, adenovirus, coronavirus (HKU1, NL63, OC43 and 229E), *Chlamydophila pneumoniae* and *Mycoplasma pneumoniae*. The atypical bacterial pathogens *C. pneumoniae* and *M. pneumoniae* were excluded from the analysis.

b Two patients had more than one respiratory virus detected (one patient with human metapneumovirus and enterovirus/rhinovirus; 1 patient with human metapneumovirus, respiratory syncytial virus A and coronavirus 229E).

**Table 5:** Nine patients with additional respiratory viruses detected only in their saliva for the second cohort of patients

**Case Number**	**Sex/age in years**	**Underlying medical conditions**	**Presenting symptom; final diagnosis**	**Respiratory virus detected in NPA**^a^	**Additional virus detected in saliva**^a^	**Potential changes in antiviral treatment or infection control practice**
1	M/56	Nasopharyngeal carcinoma	Productive cough, dyspnea; pneumonia	Negative^b^	Influenza A H1	Neuraminidase inhibitor
2	F/78	Gout	Fever, dyspnea; lower respiratory tract infection	Negative^b^	Influenza A H3	Neuraminidase inhibitor
3	M/81	DM, Ca prostate	Productive cough; upper respiratory tract infection	Negative^c^	Influenza A H3	Neuraminidase inhibitor
4	F/81	Hypertension, hyperlipidemia, IFG	Rhinorrhea, cough, dyspnea, wheeze; pneumonia	EV/RV^b^	hMPV	Contact precaution
5	M/72	DM, hypertension	Fever, rhinorrhea, dry cough; pneumonia	Negative^b^	hMPV	Contact precaution
6	M/55	Schizophrenia, gout, CVA	Fever, productive cough; pneumonia	Negative^c^	hMPV	Contact precaution
7	M/63	Asthma, Churg-Strauss syndrome	Productive cough, dyspnea; asthmatic attack	Negative^b^	EV/RV	Nil
8	F/63	Stage IV lymphoma, hypertension, asthma	Fever, productive cough, chills; upper respiratory tract infection	Negative^b^	EV/RV	Nil
9	M/79	Ischemic heart disease, diabetic nephropathy, Ca prostate with bone metastasis, pemphigoid, bronchiectasis	Fever and dizziness; CAPD peritonitis	Negative^c^	Coronavirus OC43	Nil

Abbreviations: carcinoma, Ca; continuous ambulatory peritoneal dialysis, CAPD; cerebrovascular accident, CVA; diabetes mellitus, DM; enterovirus/rhinovirus, EV/RV; human metapneumovirus, hMPV; impaired fasting glucose, IFG.

a Respiratory virus detection was performed using NxTAG Respiratory Pathogen Panel.

b Sufficient nasopharyngeal columnar epithelial cells detected during DFA.

c Insufficient nasopharyngeal columnar epithelial cells detected during DFA.
