# Supplementary material for: Additional molecular testing of saliva specimens improves the detection of respiratory viruses
Source: Emerg Microbes Infect. 2017 Jun 7;6(6):e49–. doi: 10.1038/emi.2017.35 (PMC5520312; doi:10.1038/emi.2017.35)
Supplement: Supplementary Table S1 [file emi201735x1.doc]

**Supplementary Table S1. Clinical characteristics of the patients in this study**

| Characteristics | Patients with respiratory viruses detected in NPA specimens during routine clinical tests^a^ (n=159) | Patients with respiratory viruses not detected in NPA specimens during routine clinical tests (n=99) |
| --- | --- | --- |
| Median age in years No. (range) | 69 (20-98) | 72 (21-93) |
| Female No. (%) | 86 (54.1) | 37 (37.4) |
|  |  |  |
| Underlying conditions No.(%) |  |  |
| Hypertension | 67 (42.1) | 50 (50.5) |
| Chronic cardiac disease | 43 (27.0) | 25 (25.3) |
| Chronic pulmonary disease | 28 (17.6) | 26 (26.3) |
| Chronic hepatic disease | 9 (5.7) | 9 (9.1) |
| Chronic renal disease | 17 (10.7) | 10 (10.1) |
| Neurological conditions | 22 (13.8) | 21 (21.2) |
| Diabetes mellitus | 30 (18.9) | 34 (34.3) |
| Connective tissue disease | 7 (4.4) | 4 (4.0) |
| Solid organ malignancy | 15 (9.4) | 21 (21.2) |
| Hematological malignancy | 20 (12.6) | 7 (7.1) |
|  |  |  |
| Charlson comorbidity score  No. (%) |  |  |
| 0 | 53 (33.3) | 22 (22.2) |
| 1 | 38 (23.9) | 22 (22.2) |
| 2 | 33 (20.8) | 25 (25.3) |
| 3 | 17 (10.7) | 20 (20.2) |
| 4 | 9 (5.7) | 1 (1.0) |
| 5 | 5 (3.1) | 3 (3.0) |
| 6 | 3 (1.9) | 1 (1.0) |
| ≥7 | 1 (0.6) | 5 (5.1) |

^a^ Routine clinical testing was performed using antigen detection by direct immunofluorescence assay (DFA), which included influenza A and B viruses, parainfluenza virus types 1-3, respiratory syncytial virus, human metapneumovirus and adenovirus. From March 1 to April 8, 2015 (during the peak season of influenza A virus), monoplex real-time reverse transcription polymerase chain reaction (RT-PCR) for influenza A M gene was performed for patients admitted to the general medical ward.
